# Supplementary material for: Barriers to the hospital treatment among Bede snake charmers in Bangladesh with special reference to venomous snakebite
Source: PLoS Negl Trop Dis. 2023 Oct 2;17(10):e0011576. doi: 10.1371/journal.pntd.0011576 (PMC10545105; doi:10.1371/journal.pntd.0011576)
Supplement: S2 File — (DOCX) [file pntd.0011576.s002.docx]

**S2. Consolidated criteria for reporting qualitative studies (COREQ): 32-item checklist**

| **Domain 1: Research team and reflexivity** | | | |
| --- | --- | --- | --- |
| Personal characteristics | | | |
| *1.* | Interviewer/facilitator | Which author/s conducted the interview or focus group? | Ken Yoshimura |
| *2.* | Credentials | What were the researcher's credentials? *E.g.*  *PhD, MD* | Master of Public Health |
| *3.* | Occupation | What was their occupation at the time of the study? | Researcher |
| *4.* | Gender | Was the researcher male or female? | Male |
| *5.* | Experience and  training | What experience or training did the researcher have? | He has been working snake/snakebite research projects. He has reviewed a manuscript for publication in PLOS Neglected Tropical Diseases. |
| Relationship with participants | | | |
| *6.* | Relationship  established | Was a relationship established prior to study commencement? | Yes, they visited the community and persevered to build a good relationship with participants |
| *7.* | Participant knowledge of the interviewer | What did the participants know about the researcher? *E.g. Personal goals, reasons for* *doing the research* | Participants were explained the research objectives and were aware their answers may contribute to help their situation. |
| *8.* | Interviewer characteristics | What characteristics were reported about the interviewer/facilitator? *E.g. Bias, assumptions,*  *reasons and interests in the research topic* | Participants were explained principal investigator’s background and the reason why he was interested in the research topic |
| **Domain 2: Study design** | | | |
| Theoretical framework | | | |
| *9.* | Methodological orientation and theory | What methodological orientation was stated to underpin the study? *E.g. grounded theory, discourse analysis, ethnography,*  *phenomenology, content analysis* | Conventional content analysis |
| Participant selection | | | |
| *10.* | Sampling | How were participants selected? *E.g. purposive,*  *convenience, consecutive, snowball* | Snowballing sampling |
| *11.* | Method of approach | How were participants approached? *E.g. face-*  *to-face, telephone, mail, email* | With the support of Bede leader, participants were introduced by snowballing sampling |
| *12.* | Sample size | How many participants were in the study? | Sixty-four participants |
| *13.* | Non-participation | How many people refused to participate or  dropped out? What were the reasons for this? | Six non　venomous snakebite victims were excluded as these individuals would be aware that the bites of these snakes are non venomous and no health seeking behavior is required.    - |
| Setting | | | |
| *14.* | Setting of data  collection | Where was the data collected? *E.g. home, clinic,*  *workplace* | Private office or at the participant’s house. |
| *15.* | Presence of non-  participants | Was anyone else present besides the  participants and researchers? | Yes, two research assistants |
