# Supplementary material for: Barriers to the hospital treatment among Bede snake charmers in Bangladesh with special reference to venomous snakebite
Source: PLoS Negl Trop Dis. 2023 Oct 2;17(10):e0011576. doi: 10.1371/journal.pntd.0011576 (PMC10545105; doi:10.1371/journal.pntd.0011576)
Supplement: S3 File — (DOCX) [file pntd.0011576.s003.docx]

S3 Informed Consent Form

**Informed Consent Form (for Interview)**

Principal Investigator: Ken YOSHIMURA MPH student from Nagasaki University, Japan

If you do NOT understand any part of this informed consent, please ask the investigator.

Your participation is voluntary though you may decide not to participate in the research at all

and also have the right not to answer any question or may withdraw any time from the

research even after giving consent. Moreover, you can answer orally according to your condition.

Objective: To clarify the health seeking behavior of Bede people for snake bite and its outcome

in Bede village in Bangladesh.

Procedure: We will ask some questions about your snake bite episode and basic information.

Confidentiality: Your identity will remain strictly confidential. But the result may be accessed by representatives of the authorities supporting this study. If you are willing to participate in the interview, then sign your name below.

For further information If you want to know more about this research project, please contact

Mr. Ken YOSHIMURA: Tel: Mail-address: kenyoshimura5041@outlook.jp

Agree/disagree (circle) Date _______/_______/2020

Participant name and signature _____________________ / ________________

Appendix A-2 Interview Guide for snake bite healer
