# Supplementary material for: Barriers to the hospital treatment among Bede snake charmers in Bangladesh with special reference to venomous snakebite
Source: PLoS Negl Trop Dis. 2023 Oct 2;17(10):e0011576. doi: 10.1371/journal.pntd.0011576 (PMC10545105; doi:10.1371/journal.pntd.0011576)
Supplement: S4 File — (DOCX) [file pntd.0011576.s004.docx]

S4 Interview guide for snakebite healer

**INTERVIEW GUIDE FOR SNAKE BITE HEALER** Date / /

| Sex | Male           Female         Other (Hijra) |
| --- | --- |
| Age | Years old  （10s・20s・30s・40s・50s・over　60s） |
| Religion | Islam  Hinduism Buddhism Christianity |
| Education (what is the highest level of education you completed?) | Never gone to school  Primary school completed  Secondary school completed  Over secondary school completed  Other (             ) |
| Hometown (Birthplace) |  |
| You live as a nomadic Bede or settled down? | Nomad  Settled |
| How long have you been living in this area? |  |
| Where do you work usually?  Do you have any specific community to serve?  (ask annual moving route if any) |  |
| Regarding snake bite care | |
| How long have you been working as a snake bite healer? | (          ) years |
| Do you have a second job?  (If so, what is the job?) | Yes (                 )  No |
| Why did you choose this job (snake bite healer)?  What made you decide to get this job? |  |
| Do you have relatives who have the same job? |  |
| Have you ever received any training?  (if “yes”)  ・How long?  ・Where?  ・What kind of training? |  |
| How many clients come in a day? |  |
| What do you get for the treatment from clients?  (if “money”) how much? |  |
| Monthly income | Tk |
| How do patients know about your treatment? |  |
| Do you feel your job demand has decreased recently? | Yes  No |
| How do you provide the care to your clients?  What sort of method and technique?  What kind of tools do you use during the treatment? |  |
| How does your treatment work to your client? |  |
| Have you ever used anti-venom? |  |
| What do you think about your mission for this job? |  |
| Have you ever talked Community Health Worker about snake bite patients? |  |
| Have you ever referred to the clients to the hospital? |  |
| What do you think about Western biomedicine? (= Allopathy) |  |
| Are there any people who died from snake bite? (including your clients)  　　 →If so, how many?  Are there any people who were dramatically recovered from snake bite? (including your clients)   →If so, how many?  Ask about the five most recent cases.  Ask the detail.   　　　　　↓  If there are above case, **Proceed the INTERVIEW GUIDE TO A PERSON BITTEN BY A SNAKE** |  |
