# Supplementary material for: Barriers to the hospital treatment among Bede snake charmers in Bangladesh with special reference to venomous snakebite
Source: PLoS Negl Trop Dis. 2023 Oct 2;17(10):e0011576. doi: 10.1371/journal.pntd.0011576 (PMC10545105; doi:10.1371/journal.pntd.0011576)
Supplement: S5 File — (DOCX) [file pntd.0011576.s005.docx]

S5 Interview Guide to a person bitten by a snake (Bede snake charmer)

**INTERVIEW GUIDE For Snakebite Victim** Date      / /

**(When interviewing to OTHER PERSON’S CASE, such as in the case of a dead person,**

**the following question “you “regard as the person in the case.)**

| Experiences of snake bite and care(health) seeking behavior | |
| --- | --- |
| Where  (place the person was bitten by a snake) | water, field, roadside, inside home, home premises  Other( ) |
| When  (which season,) | Summer, dry season, rainy season other(           ) |
| What time |  |
| Bite site |  |
| What kind of snake? (snake identity) with pictures |  |
| Signs and Symptoms |  |
| What were you doing when you were bitten by a snake? |  |
| How did you take care of it?  ex. self-treatment,  go to a hospital,  anti-venom,  Bede’s folk medicine. |  |
| Why did you choose the way? |  |
| Who was involved in the decision the way? |  |
| Did you take care of it immediately?  If not, when did you take care of it?  Why did not you respond immediately? |  |
| Do you have the aftereffect of the snake bite?   If so, what kind of? |  |
| Are/were there any changes in your life after snake bite?   If yes, what are/were those changes? |  |
| Did you lose something after the snake bite?   If so, why do you think you lost them? |  |
| Are you satisfied with the care for the snake bite? |  |
| What do you think is the best way to care for snake bite? |  |
| Why do you think so? |  |
| Have you ever gotten a vaccination for tetanus?  (if the person has EPI card, please check it) |  |
| How many times have you been bitten by a snake?  →If the person has multiple experiences, ask the details as well.  Ask the reason why the person was bitten multiple times (agriculture, flood, snake-handling, etc.). |  |
| What barriers do people face getting care for snake bite? (Distance, cost, culture, etc.) |  |
| Tell me what the best way is to provide information about snake bite and its services? |  |

| Basic information | |
| --- | --- |
| Sex | Male           Female         Other (Hijra) |
| Age | Years old  （10s・20s・30s・40s・50s・over　60s） |
| Religion | Islam  Hinduism Buddhism Christianity |
| Education (what is the highest level of education you completed?) | Never gone to school  Primary school completed  Secondary school completed  Over secondary school completed  Other (           ) |
| Hometown (Birthplace) |  |
| Do you live as a nomadic Bede or settled down? | Nomad  Settled |
| The number of earning member in your family |  |
| Occupation |  |
| Where do you go around  (moving route) |  |
| Have you ever suffered from diseases/injury?  (Medical history) | Filariasis, Kala-azar (Leishmaniasis), Dengue fever, Major injury, Occupational hazard  Diarrhea, Respiratory diseases, Fever,  Others (                                       ) |
| Monthly income | (             ) Tk |
| Have you ever felt stigma or prejudice as being a Bede?  (If so, ask the detail) | Yes  No  The detail |
