# Supplementary figures and images for: Barriers to the hospital treatment among Bede snake charmers in Bangladesh with special reference to venomous snakebite
Source: PLoS Negl Trop Dis. 2023 Oct 2;17(10):e0011576. doi: 10.1371/journal.pntd.0011576 (PMC10545105; doi:10.1371/journal.pntd.0011576)

S6 Field photos

Photo 1. Herbal medicine


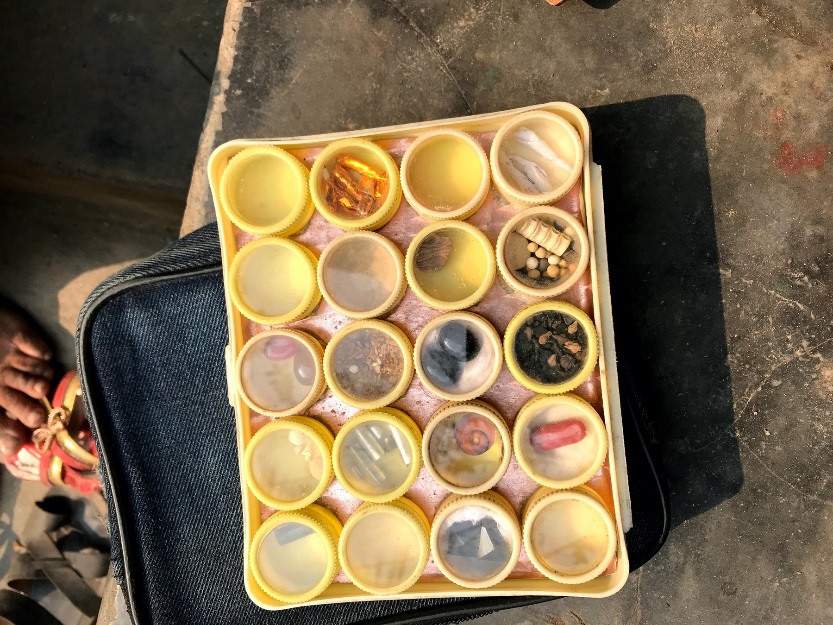


Photo 2. Snake show


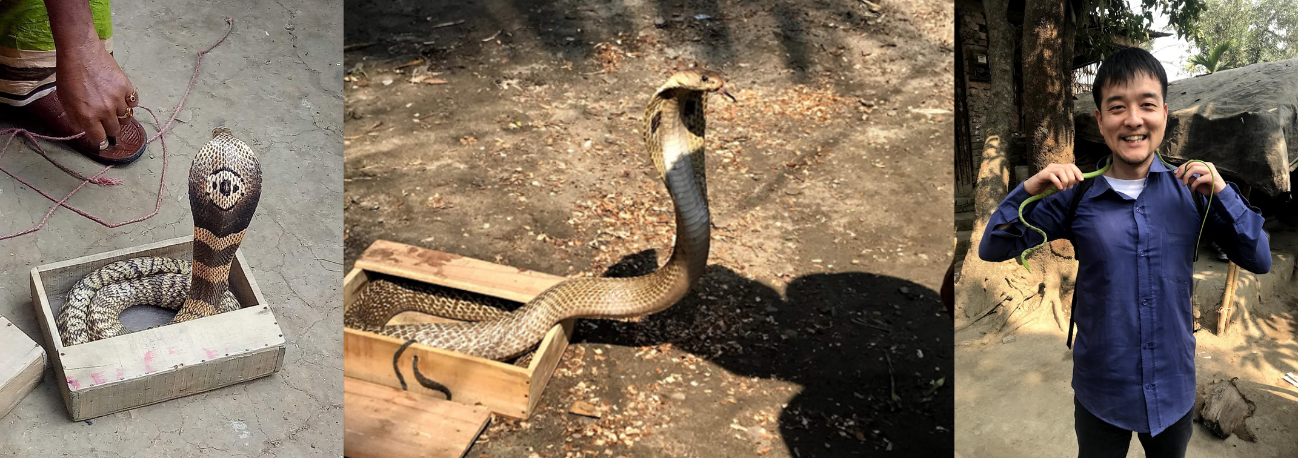

Supplement: S6 File — (DOCX) [file pntd.0011576.s006.docx]
