# Supplementary material for: Barriers to the hospital treatment among Bede snake charmers in Bangladesh with special reference to venomous snakebite
Source: PLoS Negl Trop Dis. 2023 Oct 2;17(10):e0011576. doi: 10.1371/journal.pntd.0011576 (PMC10545105; doi:10.1371/journal.pntd.0011576)
Supplement: Attachment — Submitted filename: Ken Yoshimura response letter 05MAR23.docx [file pntd.0011576.s007.docx]

AUTHOR RESPONSE LETTER

PNTD-D-22-01571: Barriers to the hospital treatment among Bede snake charmers in Bangladesh with special reference to venomous snakebite

Dear Editor,

On behalf of all the authors, I would like to resubmit our manuscript entitled: “Barriers to the hospital treatment among Bede snake charmers in Bangladesh with special reference to venomous snakebite” (PNTD-D-22-01571).

The comments forwarded from the editor and reviewers are highly valued and

contain useful suggestions. We thoroughly revised our manuscript and addressed each concern raised by the reviewers.

We express our gratitude for the constructive comments from the reviewers and made every effort to incorporate them in this updated version.

All authors have contributions meet the criteria for authorship and all authors have

signed a declaration confirming authorship, disclosing any potential conflicts of interest, and transferring the copyright if the manuscript is accepted for publication.

We certify that this work is original and has not been previously published or submitted for publication elsewhere in any language.

Thank you for considering this paper for publication. Sincerely yours,

Ken Yoshimura Japan Snake Institute

3318 Yabuzukah-machi, Ota-shi, Gunma-ken 379-2301 Japan Email: [yoshimura@snake-center.com](mailto:yoshimura@snake-center.com)

Tel: +81-277-78-5193

# REVIEWER’S COMMENTS

**Methods**

*Reviewer #1: The title mentioned an epidemiological survey on snakebites. The methodology is actually qualitative research that uses the snowball method to recruit interviewees within the Bede nomadic community and describe their barriers to seek hospital treatment for snakebite envenoming. I don't see methods relevant for an epidemiological survey and no results that would describe the incidence of snakebites within this particular community.*

**Response**: Thank you for your valuable comments. We agree with your comments. We have revised the title from “Barriers to the hospital treatment among Bede snake charmers in

Bangladesh: epidemiological survey of snakebite” to “Barriers to the hospital treatment among Bede snake charmers in Bangladesh with special reference to venomous snakebite”, taking into account the suggestions from other reviewers as well.

| Original version | Revised version |
| --- | --- |
| Barriers to the hospital treatment among Bede snake charmers in Bangladesh: epidemiological survey of snakebite | Barriers to the hospital treatment among Bede snake charmers in Bangladesh with special reference to venomous snakebite |

Reviewer #2:

*Comment 1: The objective of the study, to “clarify the health seeking behavior of the Bede people regarding snakebite and its outcome”, is clearly articulated in the penultimate sentence of the Introduction. The stated hypothesis, that the study findings should “improve the situations of snakebite among the Bede people as well as…the public health situation in Bangladesh” is testable, but the testing of that hypothesis was not an aim of this study.*

**Response**: We highly appreciate your comments and agree with your comments. We revised our strong conclusion of “improve the situations of snakebite among the Bede people as well as…the public health situation in Bangladesh” to “help to overcome barriers for the hospital treatment and provide insights into the health seeking behavior of the Bede people regarding snakebite” (page 7, line 138-141).

| Original version | Revised version |
| --- | --- |
| improve the situations of snakebite among the Bede people as well as…the public health situation in Bangladesh | help to overcome barriers for the hospital treatment and provide insights into the health seeking behavior of the Bede people regarding snakebite |

*Comment 2: As a qualitative work, the study design was appropriate to address the stated objective. The selection process of individuals to be interviewed was vague, but my*

*understanding is that the researchers employed snowball sampling to recruit a total of 64 interviewees of the Bede people: 38 snakebite charmers who had survived a venomous bite in the previous 3 years, 6 relatives of snakebite charmers who had been bitten in the previous 3 years but had not survived, and 20 snakebite traditional healers. This selection process should be stated more precisely and succinctly. The 64 interviewees were appropriate, given the aim of the study.*

**Response**: Thank you for your insightful comments. Your understanding is indeed correct. Our recruitment process was based on snowball sampling, which resulted in 64 total interviewees, including 38 snakebite charmers who survived a venomous bite in the last 3 years, 6 relatives of deceased charmers, and 20 traditional healers. We regret not expressing this more accurately in the paper. To clarify the selection process for readers, we have added Figure 2 "recruitment chart of participants" (page 10, line 196- 197, Fig2, recruitment chart of participants).

*Comment 3: No statistical analysis was performed or required. On page 9, line 169: neither the paper nor the reference provides sufficient proof that the snakebite victims in this study had experiences “sufficiently traumatic to leave a vivid memory for many years”. This declaration should either be removed or reworded to indicate that the authors presume that the snakebite victims’ experiences were sufficiently traumatic to leave a reliable memory years after the event.*

**Response**: Thank you again for your constructive comments. We totally agree with your comments. We have removed the declaration “Their experiences are sufficiently traumatic to leave a vivid memory for many years [21]” (page 9, line 179-180) since this is not critical to the main argument.

*Comment 4: Regarding ethical or regulatory concerns, lines 191-192, “When the participants could not sign their names on the document, we obtained verbal consent from their families”, leave one to wonder why the participant could not give his own consent—unless here we are referring only to deceased victims, in which case this should be stated.*

**Response**: We apologize for the confusion in the previous sentence. We initially thought that some of the participants were illiterate, but it turned out that no one who was illiterate participated. All participants, including the family of the deceased, were able to sign their names. Hence, we deleted the sentence “When the participants could not sign their names on the document, we obtained verbal consent from their families” (page 11, line 211-212).

*Comment 5: Also, what does it mean that “IACIB…offered the Bede free health care”? Free comprehensive health care in perpetuity? During the course of the study? For snakebite treatment only?*

**Response**: Thank you very much for your question. The IACIB, with a hospital in Savar, has a history of offering free health care services to the Bede community, including conducting

situation analysis and supporting research efforts. The IACIB conducted free health care services once before the course of the study not only for snakebite treatment but for all health concerns.

From 2014 to 2019, the IACIB organized multiple free health camps in the community with support from various organizations, such as Marie Stopes, SNV-The Netherlands Development organization, Alliance UK, and the Ministry of Health and Family Welfare, Government of Bangladesh. During these camps, a team of medical doctors, nurses, and paramedics provided consultations, collected diagnostic samples, treated patients, and referred complicated cases to nearby hospitals, all free of charge. The camps were conducted once or twice a week and the local community was alerted through loudspeaker announcements on the preceding day.

We deleted the previous sentence and added the following concise sentence “

“IACIB, which has a hospital in Saval, provided its support to conduct this research. The organization has a history of providing free health care services to the Bede community, including snakebite treatment, from 2014 to 2019 with support from various organizations.” (page 11, line 213-218)

| Original version | Revised version |
| --- | --- |
| IACIB, which has a hospital in Savar, offered the Bede free health care services and conducted the situation analysis in the past, provided its support to conduct this research. | IACIB, which has a hospital in Saval, provided its support to conduct this research. The organization has a history of providing free health care services to the Bede community, including snakebite treatment, from 2014 to 2019 with support from various organizations. |

*Reviewer #3: Study is appropriate for the population of interest. Sample size is small but expected. No ethical concerns are present and research team utilized appropriate methods for informed consent and institutional approval.*

**Response**: Thank you for your time and consideration in reviewing our manuscript.

# Results

*Reviewer#1*

*Comment 1: In table 2: symptoms: do the authors have any information about symptoms (neurotoxic signs or bleeding) in the group of survivors.*

**Response**: We are thankful for your constructive criticisms. We have added information about symptoms (neurotoxic signs or bleeding) in the group of survivors in table2 (page 16, line 283- 284). The results indicate that most of the victims in the group of survivors were bitten by Elapidae snakes, which typically cause neurotoxic symptoms. However, a significant number of survivors also experienced bleeding symptoms.

*Comment 2: Under treatment it is mentioned that 30 snakebite victims had treatment at a hospital, but under Snakebite coping and in Figure 2 there are only 5 patients seeking care in a hospital and the majority went for traditional treatment or no treatment.*

**Response**: Thank you for your valuable comment and we apologize for lack of explanation. We've corrected the mistake in Table 2 and changed "Treatment" to "Best Treatment" in table 2 (page16, line 283-284). *“Under the treatment in table 2, it is mentioned that 30 snakebite victims had treatment at a hospital”* means we asked the participants “What do you think is the best way to care for snake bite? and 30 among the 38 participants answered “hospital treatment is the best way to care for snakebite (page13, line 247). The 30 snakebite victims mean the number of the participants who answer, “the best treatment is at a hospital”, not actual number of visiting a hospital for the treatment. Under snakebite coping of the table 2, there are only 5 patients means actual number of visiting hospital, and the number of the 5 of the figure means the actual number of visiting hospital, as well.

| Original version | Revised version |
| --- | --- |
|  |  |

*Comment 3: In table 1 the authors mention income within the group of 38 survivors. It would be interesting to have numbers about the income within the whole Bede population of 1.2 million and for Bangladesh. Is there a difference?*

**Response**: Thank you for your valuable comment. At this time, we are unable to determine whether there is a difference in income between the group of 38 survivors and the whole Bede population of 1.2 million as a whole.

*Reviewer #2*

*Comment 1: The results are clearly presented on the whole, but there are problems.*

*To be consistent and to improve comprehension, the heading on page 11 line 209 should have “survivors” in parentheses after “snake charmers” since the subsequent heading has “deceased cases” in parentheses.*

**Response:** We appreciate your valuable comments. We have added “survivors” in parentheses after “snake charmers” (page 12, line 240).

*Comment 2: On page 12, line 230, why is this statement inserted here and not under the section labeled Barriers…?*

**Response**: We thank you for your insightful comments. Because we thought it is necessary to give overview of the results in Table 2 in this section, which looks logically. However, we have inserted the statement on the section labeled Barriers as well (page 24, line 455-457).

*Comment 3: I do not understand the sentence on lines 240-241 of page 12. If I assume that the intention is to say that the individual with full secondary school education was excluded from analysis, I then must ask, “What analysis?” We see the more highly educated person included in Figure 3. We do not see any other analysis.*

**Response**: We acknowledge and appreciate your feedback. After considering the comment from other reviewer, we have removed Figure 3 and the corresponding sentence. (page13, line 260- 262 and page 18, line 291-292.)

*Comment 4: Table 2. Is there a contradiction in stating that 5 of the 6 death cases occurred during a performance (page 12, line 245), yet in the table, 1 occurred roadside and 4 in the field? Were all these occurrences during performances?*

**Response**: We appreciate your insightful remark. All those were not occurrences during performances. Five cases occurred during a snake charming performance while the reminder: one case occurred during feeding in the room. We mentioned in the table2, 1 occurred roadside, 4 in the field and 1 in the room, which is feeding.

*Comment 5: Table 2. There seems to be a contradiction in Table 2 and Figure 2 in that Table 2 says 30 survivors were treated at hospital whereas Figure 2 shows 5 (4 + 1) were treated at hospital. Can this discrepancy be clarified?*

**Response**: Thank you for your valuable comment and we apologize for lack of explanation. We've corrected the mistake in Table 2, page16 and changed "Treatment" to "Best Treatment" in table2.

*“Under the treatment in table 2, it is mentioned that 30 snakebite victims had treatment at a hospital”* means we asked the participants “What do you think is the best way to care for snake bite? And 30 among the 38 answered “the best treatment is treatment at a hospital (page13, line 247-248). The 30 snakebite victims mean the number of the participants who answer “the best treatment is at a hospital”, not actual number of visiting a hospital for the treatment.

Under snakebite coping of the table 2, there are only 5 patients means actual number of visiting hospital, and the number of the 5 of the figure means the actual number of visiting hospitals, as well.

| Original version | Revised version |
| --- | --- |

|  |  |
| --- | --- |

*Comment 6: Table 2. Why is the cell that counts symptoms (neurotoxic vs. NA) empty in the column of Survival cases?*

**Response**: Thank you for pointing out mistakes. We have added information about symptoms (neurotoxic signs or bleeding) in the group of survivors in table2 also considering other reviewer’s comment. The results indicate that most of the victims in the group of survivors were bitten by Elapidae snakes, which typically cause neurotoxic symptoms. However, a significant number of survivors also experienced bleeding symptoms

*Comment 7: Figure 3. The title should insert the word “victims” between “snakebite” and “and”—assuming that this is what this graph is depicting. But does this figure have any real value? Without a control group, we can have no idea whether there is a relevant correlation between education and snake charmers who have survived a snakebite. Would any randomly chosen characteristic of the Bede (odd-number year of birth, for example) give a similar distribution of education level?*

**Response**: Thank you for your valuable comments. After considering the comment from other reviewer, we have removed Figure 3 and the corresponding sentence. (page13, line 260-262 and page 18, line 291-292).

*Comment 8: The section describing health-seeking behaviors is of great interest, and the use of verbatim quotations is valuable.*

**Response**: Thank you for your time and consideration in reviewing our manuscript.

*Reviewer #3: Results are clearly presented. Table and figures are sufficient. I think a map is important. One suggestion would be to add population density and other geographical features of the study site. Another suggestion would be to add a figure of the four venomous species which the snake charmers were bitten by. Added to line 224. Seeing these snakes helps the readers have a visual understanding of the species and possibly the risks of a snake charmer.*

**Response**: Thank you for your valuable comments and positive feedback of results. We agree with your comments. While we have added the figure of the four venomous species, we are afraid that we could not obtain the information of the density and other geographical features of the study site (page 14, line 265-269, Fig3.The four venomous species which the snake charmers were bitten by)

# Conclusions

*Reviewer #1*

*Comment 1: The authors conclude in the ABSTRACT that the investigation of the health seeking behaviour of the Bede snake charmers may contribute to the improvement of the public health situation in Bangladesh. The Bede are just 1.2 Million of 166 Million people in Bangladesh and they are obviously a special group that earn their income through snake related occupations. It is questionable to infer the entire population from the small Bede community.*

**Response**: Your input has been extremely valuable. The investigation of the health-seeking behavior of the Bede snake charmers may contribute to the improvement of snakebite treatment in Bangladesh. Our study focused on the treatment practices of the Bede community, who are the primary source of treatment for snakebite victims in the country. Hence we deleted the sentence “These results suggest that the investigation of the health-seeking behavior of the Bede snake charmers may contribute to the improvement of the public health situation in Bangladesh” and added “Our results provide that insights into the current state of snakebite treatment in Bangladesh and can inform the development of more effective and accessible treatment options for those affected”. (page 3, line 50-53)

| Original version | Revised version |
| --- | --- |
| These results suggest that the investigation of the health-seeking behavior of the Bede snake charmers may contribute to the improvement of the public health situation in Bangladesh. | Our results provide that insights into the current state of snakebite treatment in Bangladesh and can inform the development of more effective and accessible treatment options for those affected. |

*Comment 2: The conclusion at the end of the discussion is very confusing: The Bede’s traditional treatments are less effective, although, Indian snakeroot may be effective. From where did the authors get this information and the conclusion that consequently few Bede people die and many suffer from severe consequences? Do the authors have any data on mortality and morbidity in the Bede Community?*

**Response**: Thank you for your valuable comments.

We have deleted the sentence” although, Indian snakeroot may be effective” and added “while many of them rely on their traditional treatment, specially their herbal medicine " (page 33, line 728-729)

| Original version | Revised version |
| --- | --- |
| although, Indian snakeroot may be effective. | while many of them rely on their traditional treatment, specially their herbal medicine |

We don’t have mortality rate and morbidity rate, but have the following verbatim quotations related to mortality and morbidity.

On page 19, line 316-318, Snakebite incidence is high in this place (Pura village) because we go to many places and return here with snakes we caught. So sometimes snakes bite us, when we are playing or feeding (Snake Charmer, 30s, male).

On page 20, line 332-334

In the festival, we intentionally get bitten by snakes on a stage, in front of other Bede groups, in order to show our power and superiority in handling snakebite (Snake Charmer 40s male).

On page 22, line 411-412

I was bitten by a snake over 70 times. but I don’t have any problems (snake charmer 60s male). On page 29, line 615-616

A lot of people have died before, but even now two, three people among us have died because of snakebite every year (Ojha ,20s, female).

On page 29 line 621-622

My uncle, grandfather, other relatives died from snakebite. Just six months ago, one neighbor, a young boy, died (Snake charmer male 30s).

*Comment 3: Maybe the risk for snakebite envenoming in the Bede Community is even lower compared to the general population, because they are experienced in snake handling and removed the fangs and even the venom glands in many snakes they used during their snake charming performance.*

**Response**: We appreciate your insightful remarks. We fully concur with your point of view. We added the following sentence in Discussion (page,31 line,677-681)

The Bede’s expertise in snake charming and the practice of removing the fangs and venom glands may play a role in reducing the risk of snakebite envenoming, as their snakebite envenoming rate may be lower compared to the general population.

However, there are still instances of snakebite-related fatalities among the Bede community each year, as evidenced by six unfortunate deaths found in this study.

*Reviewer#2*

*Comment 1: Some of the conclusions are supported by the data, but others are not. The final section of the Discussion claims that early, appropriate treatment is essential for the treatment of*

*snakebite. This study was not a quantitative, controlled study. It did not address or test this question, and therefore the researchers have no basis on which to draw this conclusion.*

**Response**: Thank you for your valuable comment, we totally agree with your opinion as this study did not aim to do that. We removed the sentences (page 32, line 708-717) and the references (page 38 line 875-page 40, line 894).

*Comment 2: Recall bias and selection bias are listed as the study’s two limitations. This is valid. The authors discuss how their data should be helpful to the advancement of our understanding of snakebite treatment behavior among the Bede. By drawing attention to the importance of traditional practices and beliefs in this society, the paper’s public health relevance extends, in spirit if not in detail, to other strongly traditional settings where snakebite is common.*

**Response**: Thank you for your valuable comments.

*Reviewer #3: Conclusions are supported by the collected data. Limitations addressed and some sampling bias exists. Authors discuss the importance of the findings for the population. I would suggest adding some possible interventions that could be implemented for improved awareness campaigns for Bede snake charmers and others living in this region of Bangladesh.*

**Response**: We appreciate the suggestion. We agree that improved awareness campaigns targeted towards Bede snake charmers in the region could be valuable and will consider this in future research.

# Editorial and Data Presentation Modifications?

*Reviewer #1: (No Response)*

**Response**: Thank you for your time and consideration in reviewing our manuscript.

*Reviewer #2*

*Comment1: A great deal of copy editing to correct grammatical and other linguistic errors is needed. The article is broader in scope than the title suggests. Only a small part of the paper deals with barriers to hospital treatment of snakebite among Bede snake charmers. The paper also covers barriers to traditional treatment, sociodemographic characteristics of snake charmers, preventive measures practiced by snake charmers, and methods of snakebite treatment employed by traditional Bede healers. The paper’s title should reflect the content of the work.*

**Response:** We are grateful for your insightful remarks. We agree with your comments. We have reviewed the manuscript again and edited for each point as follows, as well as changed the title from “Barriers to the hospital treatment among Bede snake charmers in Bangladesh:

epidemiological survey of snakebite” to “Barriers to the hospital treatment among Bede snake charmers in Bangladesh with special reference to venomous snakebite” also considering other reviewer’s comment.

| Original version | Revised version |
| --- | --- |
| Barriers to the hospital treatment among Bede snake charmers in Bangladesh: epidemiological survey of snakebite | Barriers to the hospital treatment among Bede snake charmers in Bangladesh with special reference to venomous snakebite |

*Comment2: On page 6, lines 92-93 and 95-96 are repetitious.*

**Response**: We appreciate the time you took to share your thoughts. We agree that there is repetition in these sentences. We have revised as following (page 5, line 97-100)

*On page 6, lines 92-93 and 95-96 are repetitious.*

| Original version | Revised version |
| --- | --- |
| However, many victims do not seek biomedical care but from traditional snakebite healers. Generally, this situation can be explained as a lack of facilities and high treatment costs at a hospital, many snakebite  victims seek care from traditional snakebite healers as their first treatment choice, | However, many victims do not seek biomedical care but from traditional snakebite healers. Generally, and globally, this situation can be explained as a lack of facilities and high treatment costs at a hospital, |

*Comment3: Similarly lines 102-104 and 105-106 are repetitious.* **Response:** We have revised as following (page 6,line 106-114) 102-104 and 105-106

| Original version | Revised version |
| --- | --- |
| These treatments can include potentially harmful approaches where an alarmingly high proportion of cases had an inappropriate use of tourniquets with incorrect pressure applied and making multiple incisions around the bite site to suck blood orally from the multiple cuts [5].  Traditional healers called Ojha (Ozha), who are mostly Bede, similarly use non- scientifically approved methods [14] | Traditional healers known as Ojha (Ozha), who are mostly Bede have been known to use potentially harmful methods such as the improper use of tourniquets and multiple incisions around the bite site for oral blood suction. In a significant proportion of cases, these methods have been used inappropriately and are not scientifically approved.[5],[14]. |

*Comment 4: On page 12, lines 245 and 250: since there were only 6 death cases, if 5 died during a performance, the 1 who did not should not be referred to as “the remainders”. If 5 victims died within a few hours after the snakebite, only 1 did not. That 1 should not be referred to as “the remaining cases” or “the victims”.*

**Response:** Thank you for pointing out our mistake. We appreciate your guidance. We have made the necessary corrections to the text as following. (page15, line 273-281)

| Original version | Revised version |
| --- | --- |
| Five cases occurred during a snake charming performance while the remainders occurred during feeding in the room. All snakes belonged to the Elapidae; two Indian cobras and four king cobras. While five victims received traditional snakebite treatment from Ojha, one victim sought no treatment. None was able to go to a hospital for the treatment. All victims showed neurotoxic symptoms such as numbness, muscle paralysis, and dyspnea. Five victims’ condition rapidly worsened and they died within a few hours after the snakebite. In the remaining cases, the victims were was alone at the time of death and no accurate time was confirmed. | Five cases occurred during a snake charming performance (one in roadside and four in the field) while the remainder occurred during feeding in the room. All snakes belonged to the Elapidae; two Indian cobras and four king cobras. While five victims received traditional snakebite treatment from Ojha, one victim sought no treatment. None was able to go to a hospital for the treatment. All victims showed neurotoxic symptoms such as numbness, muscle paralysis, and dyspnea. Five victims’ condition rapidly worsened and they died within a few hours after the snakebite. In the remaining case, the victim was alone at the time of death and no accurate time was confirmed. |

*Comment 5: There is considerable inconsistency between headings and subsequent text. A heading on page 16, line 274, is “Barriers influencing the health seeking behavior” yet it is not until page 20, line 420 that the paper begins to cite barriers to health seeking behavior.*

**Response**: We are grateful for your constructive criticism. We agree with your comments. We have revised the heading from “Barriers influencing the health seeking behavior” to “health seeking behavior for snakebite” (page 19,line 303-304)

| Original version | Revised version |
| --- | --- |
| Barriers influencing the health seeking behavior | Health seeking behavior for snakebite |

*Comment 6: Lines 278-304 present risk factors;*

**Response**: We have revised the heading from “Occupational hazard/disease” to “Risk factors (Occupational hazard/disease)” (page 19, line 308)

| Original version | Revised version |
| --- | --- |
| Occupational hazard/disease | Risk factors (Occupational hazard/disease) |

*Comment 7: lines 306-344 present preventive measures*

**Response**: We have revised the heading from “Safety measure against snakebite ” to “ Preventive measures (Safety measure against snakebite)” (page 20, line 336)

| Original version | Revised version |
| --- | --- |
| Safety measure against snakebite | Preventive measures (Safety measure against snakebite) |

*Comment 8: lines 346-382 present diagnosis*

**Response**: We have revised the heading from “Traditional venom test” to “Diagnosis (Traditional venom test)” (page21, line 376)

| Original version | Revised version |
| --- | --- |
| Traditional venom test | Diagnosis (Traditional venom test) |

*Comment9: lines 385-412 describe symptoms and treatment.*

**Response**: We have revised the heading from “Snake venom (Neurotoxicity or other)” to “Symptoms and treatment” (page 22, line 415)

| Original version | Revised version |
| --- | --- |
| Snake venom (Neurotoxicity or other) | Symptoms and treatment |

*Comment10: Lines 414-418 belong under Availability.*

**Response**: We have changed the sentence to under Availability (page26, line 521-524).

*Comment 11: Line 450 also probably belongs under Availability.*

**Response**: We have changed the sentence to under Availability as well (page 26, line 526-533)

*Comment12: Since the preceding pages discussed barriers to hospital treatment, a new heading before line 524 should be inserted to indicate that the ensuing text discusses barriers to traditional treatment.*

**Response**: We have inserted the heading “Barriers against the utilization of traditional treatment ” (page 27, line 570)

*Comment13: Page 21, line 422. “Accessibility” in public health is generally used to connote a combination of factors including geography, affordability, and availability. Although it puts a small dent in the “4 A’s”, “accessibility” in this paper is being used more in the sense of geographic accessibility and would be better labeled as such.*

**Response**: We have edited from the “Accessibility” to “(geographic) Accessibility” (page 24, line 455-457)

*Reviewer #3: (No Response)*

**Response**: Thank you for your time and consideration in reviewing our manuscript.

# Summary and General Comments

*Reviewer #1: The authors present the results of interviews with 38 snakebite victims, who survived the snakebite mostly during snake handling and 6 snakebite victims, who died after a bite. Although an epidemiological survey is mentioned in the title and the first part of the discussion, there are no results of such a survey. The barriers Availability, Accessibility, Affordability and Acceptability are common barriers for most snakebite victims in many regions in Asia and Africa and not really new or special for this certain community of snake charmers and handlers.*

**Response**: Thank you for your valuable comments. We agree with your comments.

We deleted the word of “epidemiological” from the title and the sentence “In addition, no epidemiological survey of snakebite among Bede snake charmers have been conducted. ” from the first part of the discussion (page 30, line 642-643). We believe that it is a significant discovery that among the snake charmers and traditional healers, who are relied upon heavily by snakebite victims in Bangladesh, there exist the same four barriers that are commonly seen in many snakebite patients across Asia and Africa

*Reviewer #2: This study is informative, original, and a welcome addition to our knowledge of snakebite. As written, readers’ comprehension suffers due to inadequate organization and mangled English. I hope the paper can be reworked to make it worthy of publication in PLOS.*

**Response**: Thank you for your valuable comments.

*Reviewer #3: Manuscript was well written, and investigation is sound for the population of interest. Only minor revisions or clarifications needed. One suggestion that I think is important is adding "venomous snakebite" to the title and "venomous snakebite" to other section headings on line 219, 242 and 268. I would ensure throughout the manuscript the "venomous snakebite" is added, like in table 2 and figure 2. Despite not knowing fully if an individual was "envenomated" we do know they were venomous snakes, and as such I believe this is important to highlight in these key areas of the manuscript. Six snake charmers were excluded because they sustained nonvenomous snakebites and the emphasis of this investigation is Bede snake charmers who handle and work with venomous snakes. Overall, excellent manuscript.*

**Response**: Thank you for your valuable comments. We agree with your comments. We have edited "venomous snakebite" to the title and "venomous snakebite" to other section headings
