# Supplementary material for: Barriers to the hospital treatment among Bede snake charmers in Bangladesh with special reference to venomous snakebite
Source: PLoS Negl Trop Dis. 2023 Oct 2;17(10):e0011576. doi: 10.1371/journal.pntd.0011576 (PMC10545105; doi:10.1371/journal.pntd.0011576)
Supplement: Attachment — Submitted filename: Ken Yoshimura-revised-authors-response-letter-11JUL23.docx [file pntd.0011576.s008.docx]

AUTHOR RESPONSE LETTER

PNTD-D-22-01571R1: Barriers to the hospital treatment among Bede snake charmers in Bangladesh with special reference to venomous snakebite

Dear Editor,

On behalf of all the authors, I would like to resubmit our manuscript entitled: “Barriers to the

hospital treatment among Bede snake charmers in Bangladesh with special reference to

venomous snakebite” (PNTD-D-22-01571R1).

The comments forwarded from the editor and reviewers are highly valued and

contain useful suggestions. We thoroughly revised our manuscript and addressed each concern

raised by the reviewers.

We express our gratitude for the constructive comments from the reviewers and made every

effort to incorporate them in this updated version.

All authors have contributions meet the criteria for authorship and all authors have

signed a declaration confirming authorship, disclosing any potential conflicts of interest, and

transferring the copyright if the manuscript is accepted for publication.

We certify that this work is original and has not been previously published or submitted for

publication elsewhere in any language.

Thank you for considering this paper for publication.

Sincerely yours,

Ken Yoshimura

Japan Snake Institute

3318 Yabuzukah-machi, Ota-shi, Gunma-ken 379-2301 Japan

Email: yoshimura@snake-center.com

Tel: +81-277-78-5193

Reviewer's Responses to Questions

**Methods**

Reviewer #2:

*Comment1: Lines 168-173:*

*Do you mean: …38 adult Bede (survival cases) as well as 1 relative of each of the 6 deceased Bede snake charmers who died due to venomous snakebite…? If so, can this be stated with that clarity?*

**Response**: Thank you for your feedback regarding the clarity of the participant composition in our study. We appreciate your suggestion to revise the statement to provide a clearer understanding of our sample. Based on your comment, we have revised the statement as follows:

| Original version | Revised version |
| --- | --- |
| The interviews were conducted with 38 adult Bede (survival cases) as well as family or relatives of the six deceased Bede snake charmers due to venomous snakebite, | The interviews were conducted with 38 adult Bede (survival cases) as well as one relative of each of the six deceased Bede snake charmers due to venomous snakebite. (Page ,9 line 193-194) |

*Comment2:*
*"6 nonvenomous snakebite victims were excluded…" Excluded from what number? If not from the 38, can you give us the total number of snakebite victims, the pool from which you selected your interviewees? Did you start with 44 snakebite survivors? Or with 50 snakebite victims?*

**Response**: I am afraid that the sentence may have been unclear. To provide clarification, the total number of snakebite victims in the initial sample was 44, and 6 individuals were excluded due to nonvenomous snakebites.

| Original version | Revised version |
| --- | --- |
| Six nonvenomous snakebite victims were excluded as these individuals would be aware that the bites of these snakes are nonvenomous and no health seeking behavior is required | One relative of each of the six deceased Bede snake charmers who died due to venomous snakebite. This approach allowed us to gather insights from both survivors and family members affected by snakebites within the Bede community. (page 9, line 194-196) |

Reviewer #3:

*Comment1: Objectives of study are clarified with revised submission. Description of population is clear. No ethical concerns are present.*
***Response****: Thank you for your valuable comment.*

Reviewer #4:

*Comment1: This article is not clear and clear, and seems to have great risks in ethics and morality. In short, there are major problems and they should be amended or rejected.*
**Response**:Thank you for your valuable input regarding our manuscript. We appreciate your feedback and concerns regarding the clarity and ethical aspects of our research.

In response to your comments, we have carefully reviewed and addressed these issues in the revised version of the article. We have made significant amendments to enhance the clarity of our findings, ensuring that the language and structure are more accessible to readers. Furthermore, we have taken proactive steps to ensure that ethical considerations are appropriately addressed.

Regarding ethical approval, we want to clarify that we obtained approval from the Institutional Review Boards (IRBs) of both Japan and Bangladesh. These approvals were obtained in accordance with the principles outlined in the Helsinki Declaration, which guides ethical research practices.

Additionally, we want to highlight that written informed consent was obtained from all participants involved in the study, including the Bede community members. This important step was taken to ensure the ethical conduct of our research and to protect the rights and welfare of the participants.

We believe that these measures, along with the revisions made to the manuscript, adequately address the points you have raised. However, we value your input and would appreciate any specific suggestions or concerns that you may still have. Your feedback will be instrumental in further improving the quality and integrity of our work.

Thank you once again for your valuable contribution to our manuscript.

**Results**
Reviewer #2:

*Comment1: I did not find that Figure 2, added as a clarification to explain participants in the study, was helpful. In fact, it is not readily understandable, and I would recommend it be removed.*
**Response**: Thank you for your feedback on Figure2 in our manuscript. We appreciate your input and agree that the figure should be easily understandable and helpful to readers. Based on your comment, we removed Figure2 from our manuscript and revised the sentences, which provides a clearer and more concise description of the selection process, outlining the use of snowball sampling and the specific categories of participants included in the study.

| **Original Version** | **Revised Version** |
| --- | --- |
| With the support of Bede leaders, participants were introduced by the snowballing sampling, which subject to bias due to difficulty to control sample composition, but common method for qualitative research on obscure populations [20] (Fig.2).  Those who had experienced snakebite in the last three years were invited to the study. The interviews were conducted with 38 adult Bede (survival cases) as well as family or relatives of the six deceased Bede snake charmers due to venomous snakebite. Six nonvenomous snakebite victims were excluded as these individuals would be aware that the bites of these snakes are nonvenomous and no health seeking behavior is required. Twenty Bede snakebite healers (Ojha) were also invited into the study to clarify their traditional therapeutic approach to snakebites. | With the support of Bede leaders, participants for the study were selected using snowball sampling, a common method employed in qualitative research for studying obscure populations [20]. Those who had experienced snakebite in the last three years were invited to participate in the study. The interviews were conducted with 38 adult Bede snake charmers who survived snakebites, as well as one relative of each of the six Bede snake charmers who died from venomous snakebites. This approach allowed us to gather insights from both survivors and family members affected by snakebites within the Bede community. Twenty Bede snakebite healers (Ojha) were also invited into the study to clarify their traditional therapeutic approaches to snakebite.(page 8, line 185-page 9 line197) |

*Comment2:*
*In Table 2, “Best treatment”, which you intended to be more explanatory, is meaningless as it stands. If I am understanding your answer to my comments to the original version, “Best treatment” should be replaced by “What do you think is the best treatment for snakebite?” or something to that effect.*
**Response**: Thank you for your feedback on Table 2. We appreciate your suggestion to replace the current column header "Best treatment" with a more direct question such as "What do you think is the best treatment for snakebite?" We agree that this will provide clearer and more meaningful information for both the participants and the reader. We have made the appropriate revisions to the table2 accordingly (Page16, Line296). Thank you for your helpful suggestion.

*Comment3:*
*Lines 280-281: This belongs in Methods, not Results.*
**Response**: We appreciate your feedback and agree with your suggestion. We have made the necessary revisions to move this information to the appropriate section. Thank you for helping to improve the clarity and organization of our manuscript.

*Comment4:*
*There are too many sub-headings in the Results section, and since the text that follows each sub-heading does not always adhere to what the sub-heading is signaling, I would suggest removing most of those sub-headings. Could you pare them down to three? These could be “Venomous snakebite experience of snake charmers (survivors)”; “Venomous snakebit experience of snake charmers (death cases)”; “Key informant interview comments”. In the key informant interview comments section, you could simply summarize with a line of text the one or multiple quotations that follow for each idea.*
**Response**: Thank you for your feedback regarding the subheadings in the Results section. We appreciate your suggestion to simplify the section and agree that fewer subheadings may improve the clarity of the manuscript. We revised the section to include the subheadings you proposed: “Venomous snakebite experience of snake charmers (survivors)”; “Venomous snakebite experience of snake charmers (death cases)”; and “Key informant interview comments”. We also considered your suggestion to summarize the key informant interview comments with a brief sentence. Thank you for helping us improve the clarity of our manuscript.

Reviewer #3:

*Comment1:Results are clear. Authors have addressed each comment or request from the Reviewer panel.*

**Response**: Thank you for your positive feedback on our manuscript. We appreciate your time and effort in reviewing our work, and we are glad to hear that our revisions have addressed each comment or request from the reviewer panel.

Reviewer #4:

*Comment1: It's not clear and complete. I even suspect that the author did not write this part at all, the author just laid out some information, meaningless.*
**Response**: Thank you for your clarification. We understand your concern regarding the presentation of the information in the results section. As this is a qualitative research study, it is important to provide a clear and comprehensive account of the findings. We acknowledge that the current presentation may not effectively convey the results and their significance. We carefully reviewed the subheadings and the accompanying text to ensure they accurately reflect the data and contribute to the overall understanding of the research findings. In light of your feedback, we considered reorganizing the subheadings to provide a more coherent structure and ensure that the text following each subheading is aligned with the specific topic being discussed. Additionally, we explored ways to summarize the key informant interview comments while still capturing the essential ideas and quotations. Our aim is to present the results in a meaningful and accessible manner, while adhering to the conventions of qualitative research reporting. We appreciate your input. If you have any further suggestions or specific recommendations for improvement, we would welcome them as we strive to enhance the quality of our manuscript.

**Conclusions**
Reviewer #2:

*Comment1: Likewise, I would suggest not subdividing the Discussion with sub-headings, since again the text after each sub-heading does not adhere closely enough to what is being signaled by the sub-headings.*
**Response**: Thank you for your suggestion regarding the Discussion section. We took your feedback into consideration and removed the sub-headings to ensure that the text flows smoothly and adheres closely to the main ideas presented.

Reviewer #3:

*Comment1: Data is presented in a concise manner.*
**Response**: Thank you for your comment. We appreciate your positive feedback regarding the presentation of our data.

Reviewer #4:

*Comment1: Too bad the author's description is not clear enough to support the conclusion. Even our argument that snakebite is not a tropical disease does not fit the purpose of the journal.*

**Response**: Thank you for your feedback. We appreciate your concern regarding the clarity of our description and conclusion. We have carefully revised the manuscript to improve the clarity and support of our argument. Regarding the statement about snakebite not being a tropical disease, we believe there might be a misunderstanding. We want to clarify that our argument is consistent with the widely recognized understanding that snakebite is indeed a tropical disease. Our study focuses on understanding the health seeking behavior of the Bede people, who reside in a tropical region where snakebite is a significant concern.

In addition, we received from the Editor at PLOS NTDs regarding the scope of our paper. The Editor has confirmed that our research is very much in scope and that the editorial decision is based on scope and several reviews. We apologize if our previous wording caused any confusion. We appreciate your attention to detail and assure you that we have made the necessary revisions to address this concern.

**Editorial and Data Presentation Modifications**

Reviewer #2:

*Comment1: Line 51: “Essential” is untested and unproven; I would suggest that another word be used.*

**Response**: Thank you for your feedback on our manuscript. We appreciate your suggestion to use an alternative word instead of "essential" in line 54. We agree and revised the sentence accordingly. Instead, we have used the phrase " has the potential to make a significant impact in reducing " to better reflect the current state of evidence.

Original version Revised version

| Original version | Revised version |
| --- | --- |
| Partnership between the public sector and the Bede community is essential to reduce snakebite morbidity and mortality in Bangladesh. | Partnership between the public sector and the Bede community has the potential to make a significant impact in reducing snakebite morbidity and mortality in Bangladesh. |

*Comment2: Lines 621-635 beginning with “One barrier to the utilization of herbal medicine…” and ending with “…has led to increasing snakebite mortality [29].” should be a separate paragraph.*

**Response**: Thank you for your feedback on our paper. We appreciate your suggestion to separate lines into a separate paragraph. We agree that this will improve the readability of the paper and help to emphasize the main point being made in that section. We made the necessary changes to the manuscript and ensure that it is updated accordingly.

*Reviewer #3: (No Response)*

**Response**: Thank you for taking the time to consider our manuscript.

*Reviewer #4: N/A*
**Response**: Thank you for taking the time to consider our manuscript.

**Summary and General Comments**

Reviewer #2:

*Comment1: I am grateful to the authors and editors for this revision. Most of my comments were addressed, and some changes were made that improve the paper.*
*I congratulate the authors on a well-researched, thoughtful study and encourage them to make every effort to improve it. Careful presentation to ensure optimal comprehension is important, not least because the topic of this paper is important.*

**Response**: Thank you very much for your kind words and for taking the time to review our paper. We greatly appreciate your feedback and suggestions, which have helped us to improve the quality of the manuscript. Your encouragement means a lot to us, and we will do our best to further refine the paper to ensure optimal comprehension. We agree that the topic is important, and we are committed to presenting it in the most effective way possible. Thank you again for your valuable contribution to the development of our paper.

*Reviewer #3:*

*Comment1: Overall, the manuscript is well written and revised manuscript is presenting interesting findings. Authors have addressed each Reviewer's comment or recommendation. In my opinion the manuscript is ready for publication with some minor editorial or grammatical corrections.*

**Response**: Thank you for your positive feedback and for considering our manuscript. We are glad that you found our revised version to be interesting and well-written. We will make sure to carefully proofread the manuscript to address any minor editorial or grammatical errors before submitting it for publication.

*Reviewer #4:*

*Comment1:There is no doubt that this manuscript pays attention to social hot issues and uses more correct research methods. However, the description of the methods is relatively thin, which is difficult for our readers to understand and reproduce, which restricts our analysis of the value of this manuscript.*

**Response**: Thank you for your comment. We understand your concern about the thin description of our methods and its potential limitations for reproducing our study. We apologize for any confusion caused and would like to clarify that the details of our study methods, including the interview guide used, are provided it as the supporting file. Regarding the snowball sampling method, we agree that snowball sampling has limitations, including the potential for bias due to its limited control over the sample composition. However, we also recognize that it is a common and effective method for qualitative research on obscure populations. We have acknowledged this limitation in our manuscript and taken steps to minimize its impact on our findings. For example, we used multiple sources to verify the information provided by the participants, and we also ensured that we had a diverse range of participants to increase the generalizability of our findings.

*Comment2: I would like to question whether the team of researchers can actually support such a study, whether other people are involved in the work but do not declare it, and whether they need to add the specific contribution of the author, preferably in detail; or why Japanese research institutions, with only a few researchers from Bangladesh, have access to primary data on Bangladesh. In which communication on Earth was the author team working at the time of the project?*

**Response:** We appreciate your question regarding the credibility and transparency of our research team. Our team consisted of researchers from Japan, Bangladesh, and Vietnam, and we declare all members who contributed to this study. Each author's specific contributions are clearly outlined as follows:

Ken Yoshimura: Conceptualization, Participants enrollment, Data collection, Data curation, Formal analysis, Investigation, Methodology, Project administration, Writing – original draft, Writing – review & editing

Moazzem Hossain: Conceptualization, Participants enrollment, Data collection, Data curation, Formal analysis, Investigation, Methodology, Project administration, Supervision, Writing – review & editing

Bumpei Tojo: Data curation, Formal analysis, Methodology, Supervision, Visualization, Writing – review & editing

Paul Tieu: Data curation, Validation, Writing – review & editing

Nathalie Nguyen Trinh: Data curation, Validation, Writing – review & editing

Nguyen Tien Huy: Data curation, Supervision, Validation, Visualization, Writing – review & editing

Miho Sato: Conceptualization, Data curation, Formal analysis, Methodology, Supervision, Validation, Writing – review & editing

Kazuhiko Moji: Conceptualization, Data curation, Formal analysis, Methodology, Project administration, Supervision, Validation, Visualization, Writing – review & editing

Regarding access to primary data on Bangladesh, we collaborated with the Institute of Allergy and Clinical Immunology of Bangladesh (IACIB), a local NGO and hospitals, to gain access to the Bede people who had experienced snakebites. Also, we revised the acknowledgement to enhance(Page,30 Line 680-681).

Our principal investigator is fluent in Bengali, enabling effective communication with the study participants.

To ensure ethical conduct and cultural sensitivity, we visited the Bede community to establish rapport and build trust. Our research was conducted with the utmost consideration for ethical guidelines and cultural norms, as detailed in our manuscript.

During the project, communication within our team primarily took place through email and in-person meetings whenever possible. This collaboration between researchers from different countries and backgrounds enhances the credibility of our findings and promotes cross-cultural understanding in research.

We hope this explanation clarifies any concerns you had regarding the composition of our team, access to primary data, and our communication methods.

*Comment3: The data came from Bangladesh. Why is it a global problem? Do people in other countries have regular contact with snakes? The expandable aspects of this study seem to have been somewhat enlarged, and it is hoped that the authors will be able to make the necessary modifications. This seems to be a "regional issue".*

**Response:** Thank you for your comment. While our study was conducted in Bangladesh, we believe that snakebite envenoming is a global problem that affects many developing countries, including those in Asia, Africa. In fact, many snakebite victims in other countries also rely on traditional healers for treatment as a first choice, as we have discussed in the introduction of our manuscript (refer to the following).
Therefore, we believe that our findings are relevant and important not only for Bangladesh but also for other countries facing similar challenges. We hope that our study will contribute to the global efforts to improve snakebite management and reduce the burden of this neglected tropical disease.

(Page 5, line 99-104)

However, many victims do not seek biomedical care but from traditional snakebite healers. Generally, and globally, this situation can be explained as “patterns of resort,”　which explains typical health-seeking behavior; that the first health seeking option tends to be the most basic and cheapest. When it is unsuccessful, people seek more advanced, costly, and unconventional treatment a lack of facilities and high treatment costs at a hospital, notably in Lao PDR [9], India [10], Nigeria [11], and South Africa [12] in terms of snakebite treatment.

[9] Vongphoumy I, Phongmany P, Sydala S, Prasith N, Reintjes R, Blessmann J. Snakebites in Two Rural Districts in Lao PDR: Community-Based Surveys Disclose High Incidence of an Invisible Public Health Problem. PLOS Neglected Tropical Diseases. 2015;9(6):e0003887. Available from: https://journals.plos.org/plosntds/article?id=10.1371/journal.pntd.0003887 [Accessed: 22 August 2022].

[10] Rao C, Shivappa P, Mothi V. Fatal snake bites – sociodemography, latency pattern of injuries. Journal of Occupational Medicine and Toxicology. 2013;8(1):7. Available from: https://occup-med.biomedcentral.com/articles/10.1186/1745-6673-8-7 [Accessed: 22 August 2022].

[11] Michael G, Thacher T, Shehu M. The effect of pre-hospital care for venomous snake bite on outcome in Nigeria. Transactions of the Royal Society of Tropical Medicine and Hygiene. 2011;105(2):95-101. Available from: https://pubmed.ncbi.nlm.nih.gov/21035155/#:~:text=Most%20(53%2C%2074%25)%20had,respectively%2C%20P%20%3D%200.02). [Accessed: 22 August 2022].

[12] Sloan DJ, Dedicoat MJ, Lalloo DG. Healthcare-seeking behaviour and use of traditional healers after snakebite in Hlabisa sub-district, KwaZulu Natal. Tropical Medicine & International Health. 2007;12(11):1386–90.

*Comment4: The author needs to adjust the logic of the article and study whether the treatment is traditional, ethnic, or modern, biotechnology-based. Sentences are a bit of a mishmash.*

**Response**:

Thank you for your comment regarding the logic of the article and the categorization of treatment methods. We appreciate your feedback and agree that it is important to clarify and categorize the different treatment approaches mentioned in the study.

In our manuscript, we have discussed various treatment methods used among the Bede people for snakebite, including herbal medicine, incision, suction, hospital treatment, and antivenom. We acknowledge that these treatment methods represent a mix of traditional, ethnic, and modern biotechnology-based approaches.

Herbal medicine, incision, and suction are commonly associated with traditional or folk remedies and can be considered traditional or ethnic treatment methods. These practices often reflect traditional healing practices within the community.

On the other hand, hospital treatment, which involves medical care provided in a hospital setting, and the use of antivenom are categorized as modern and biotechnology-based approaches. These treatments rely on medical advancements and scientific interventions to manage snakebite cases effectively. (Page10, line 212-221)

We recognize that the article could benefit from clearer categorization and logical organization of these treatment methods. In the revised version of the manuscript, we have refined the description of each treatment approach and provided a more structured and coherent presentation to improve the overall logical flow of the article.

Thank you again for bringing this to our attention, and we appreciate your valuable input in helping us enhance the clarity and coherence of our study.

*Comment5: It seems that some of the words used are too trendy for easy reading.*
**Response**: Thank you for your feedback. We apologize if some of the language used in our manuscript was difficult to read. We have taken your comment into consideration and made efforts to simplify the language and clarify any potentially confusing terms. However, we do want to note that when using quotations from our participants, we aimed to preserve their original words and phrasing to accurately reflect their perspectives and experiences. We will continue to strive for clear and accessible language while also maintaining the integrity of our participants' voices. Thank you again for your valuable input

*Comment6.In general, all data, as long as it is not obtained from one's own experiment, needs to be supported by literature sources. The source of much of the data in this manuscript is questionable, and the authors should correct it against references.*

**Response**:

Thank you for your valuable feedback on our manuscript. We appreciate your point about the importance of supporting data with literature sources. We have carefully reviewed the data presented in our manuscript and have taken the necessary steps to address the concern raised. We have attached additional literature sources that support the data presented, including a table of codes, sub-categories, and categories. These sources provide a more robust foundation for the information presented in our article. We sincerely appreciate your feedback and are committed to upholding rigorous standards in our work. Thank you for bringing this to our attention.

*Comment7: As an article published in a journal, the author of this article mentions a great deal of material unrelated to the main idea. Authors should enhance the relevance of the main idea or remove part of the content to avoid misleading readers and provide effective information. A lot of basic information is especially unnecessary.*

**Response**:

Thank you for your valuable feedback on our manuscript. We appreciate your comments regarding the inclusion of unrelated material and the need to enhance the relevance of the main idea. Also considering other reviewer’s comments, we have made revisions to some parts of the Result and Discussion of the manuscript. Specifically, we have focused on enhancing the relevance of the main idea by removing any material that was unrelated or detracted from the core message. This comprehensive approach has resulted in a more focused and cohesive manuscript.

(Page, 11 Line241 ~ Page28 Line640)

Thank you for bringing these concerns to our attention, and we are grateful for the opportunity to enhance the quality of our manuscript based on the feedback provided."

*Comment8: Does the work reported in the manuscript have relevant ethical approval? I seem to see several references to inappropriate ethical requests throughout the text. How many different institutions do the authors come from? What are the ethical requirements of other institutions? Is there a problem here? It needs to be explained.*
*The author did not control variables well. In a country with a complex religious background, many factors will affect the selection of patients and even the rehabilitation of patients in terms of psychological factors. The author appears to briefly mention these issues but does not provide any further elaboration based on them. I hope to pay attention to similar issues.*

**Response**: Thank you for raising the important question regarding ethical approval and the institutions involved in our study. We want to assure you that we have followed rigorous ethical procedures and obtained appropriate approvals to conduct this study. Ethical approval was obtained from the School of Tropical Medicine and Global Health, Nagasaki University (approval number: NU_TMGH_2020-091-0), and the National Research Ethics Committee of the Bangladesh Medical Research Council (Registration Number: 267 24 11 2019).

As the Bede community represents ethnic minorities and vulnerable groups in society, we took extensive measures to ensure ethical considerations were met. The purpose and procedures of our study were explained to their leaders, and informed consent was obtained. We also provided printed information sheets with contact details of the researchers and the Institute of Allergy and Clinical Immunology of Bangladesh (IACIB).

In terms of institutional affiliations, the authors of the manuscript come from the following eight institutions: School of Tropical Medicine and Global Health, Nagasaki University, Nagasaki, Japan; Japan Snake Institute, Gunma Prefecture, Japan; Institute of Allergy and Clinical Immunology of Bangladesh, Dhaka, Bangladesh; World Language and Society Education Centre, Tokyo University of Foreign Studies, Tokyo, Japan; McMaster University, Ontario, Canada; University of Toronto, Ontario, Canada; Online Research Club (https://www.onlineresearchclub.org/), Nagasaki, Japan; School of Global Humanities and Social Sciences, Nagasaki University, Nagasaki, Japan. The involvement of multiple institutions and their expertise has contributed to the comprehensive and interdisciplinary nature of our research. However, only two institutions including and Institute of Allergy and Clinical Immunology of Bangladesh, Dhaka, Bangladesh involving in the recruitment and interview participants. Only the PI (Yoshimura) can access the raw data and analyze it. Other authors can only give suggestion or guidance to analyze the data without accessing the raw data. Therefore, we only need IRB approval from two institution.

Regarding the control of variables, we appreciate your concern and acknowledge that various factors, including psychological factors influenced by the country's complex religious background, can impact the selection and rehabilitation of patients. While we briefly mentioned these issues in the manuscript, we understand the importance of further elaboration and exploration to gain a more comprehensive understanding. However, due to limitations in participant information, we were unable to provide extensive details in this regard. Hence, we added it on the limitation (Page 29, Line 647-654)

We agree that future research should delve deeper into these factors to enhance the understanding of their impact.

We genuinely appreciate your feedback and suggestions for improvement. If you have any specific recommendations or further queries, please feel free to share them. Your input will be valuable in enhancing the quality of our research. Thank you for your attention to these concerns.

*Comment9: There is a problem with the structure of the article. A lot of the content written in "results" should be "methods". Authors are encouraged to submit the manuscript structure to their peers in other work groups prior to submission.*

**Response**:

Thank you for your comment regarding the structure of our article. We appreciate your feedback and have carefully considered your suggestion.Upon reviewing the manuscript and taking into account the input from the peers , we agree that there was an issue with the placement of certain content within the manuscript. Specifically, we acknowledge that the sentence "Codes were from each interview and were analyzed for sub-categories that affect the health seeking behavior for snakebite" in the "Results" section would be better suited under the "Methods" section, which is also commented by other reviewer. In light of this, we have made the necessary revisions by moving the aforementioned sentence from Page 18, Line 313-314 (Results) to Page 8, Line 174-175 (Methods). This adjustment ensures a more logical and coherent flow of information within the manuscript.

*Comment10: The authors should personally attest to the appropriateness of the literature*.

**Response**: Thank you for your comment on the literature selection in our manuscript. We agree with your opinion and we have conducted a thorough review and ensured the relevance, quality, and appropriateness of the included sources. Additional literature sources, tables of codes/sub-categories/categories have been attached to strengthen the research credibility and validity.

*Comment11: The report of the results is too flat and direct, lacking logic, and easy to understand.*

**Response**: Thank you for your comment regarding the flatness and directness of the results in our manuscript. We appreciate your feedback and would like to provide some clarification.

Our study employed a qualitative research design, which aims to explore and understand the perspectives, experiences, and behaviors of individuals within a specific context. Unlike quantitative research that often presents results in a structured and statistical manner, qualitative research takes a more descriptive and narrative approach to convey the richness of the data.

Given the nature of our qualitative study, our intention was to provide an in-depth and nuanced understanding of the health-seeking behavior of the Bede people regarding snakebite. We aimed to capture their perspectives and experiences as authentically as possible, allowing the participants' voices to shine through in our findings.

While we acknowledge that the presentation of our results may appear different from quantitative studies, we believe that the narrative and descriptive style aligns with the goals and methodologies of qualitative research. It allows us to convey the complexities and nuances of the participants' experiences and provides a deeper understanding of their health-seeking behavior.

However, we take your feedback seriously and understand the importance of ensuring clarity and coherence in presenting our results. We carefully reviewed the manuscript to enhance the organization and logical flow of the findings section, making sure that the narrative is engaging and effectively conveys the key insights from our study.

*Comment12: How does this information relate to the main idea? No discourse is seen, even in the following text.*

**Response**: Thank you for your comment regarding the relevance of the information presented and the need for clear discourse in our manuscript. We appreciate your feedback and understand the importance of maintaining a clear connection to the main idea throughout the text.

Upon reviewing the manuscript, we acknowledge that there might be areas where the information could be more effectively linked to the main idea. We reassessed the organization and flow of the content to ensure a coherent and cohesive narrative that aligns with the central theme. We recognize the significance of providing a clear discourse and made the necessary revisions to strengthen the connection between the information presented and the main idea of the study. Your input is valuable in improving the clarity and effectiveness of our manuscript, and we thank you for bringing this to our attention.

*Comment13:part of the spelling problem.*
**Response**: Thank you for your comment and for bringing the spelling issue to our attention. We apologize for any errors and carefully reviewed and revised the manuscript to ensure that all spelling is correct. We appreciate your feedback and assistance in improving the quality of our work.

*Comment14:The snake-catching scene doesn't make sense. The author's survey is based on the self-description of the population concerned, and the author also mentions that they do not have a high level of education. Can they exclude the implausible or exaggerated parts of the description? No embodiment of these methods is seen. There seems to be a need for clarification.*

**Response**:
Thank you for your comment. We appreciate your feedback on our manuscript. As mentioned in the methods section, we checked the snakebite marks to ensure their credibility, which we believe helped to ensure the validity of our findings. However, we understand your concern regarding the plausibility of the snake-catching scene described in our study. Our research is based on self-reported data from individuals with varying levels of education and may therefore contain some level of exaggeration or implausibility. To address this limitation, we have added a sentence in the limitations section of our manuscript acknowledging that this study was based on self-reported snakebites and may have recall bias and inaccurate responses. We hope this clarification addresses your concerns and thank you again for your valuable input.

*Comment15: Were all the included subjects from Bangladesh? Are there exclusion criteria? It needs to be confirmed.*
**Response**: Thank you for your valuable comment. All of the subjects were from Bangladesh. We also excluded non-venomous snake bite victims, as stated in our Methods section. We hope this clarifies any confusion and addresses your concern.

*Comment16: The math and statistics seem to be wrong.*
**Response**: Thank you for your comment regarding the math and statistics in our manuscript. We have carefully reviewed the math and statistics, and we believe that the results are accurate.

*Comment17:Can you provide pictures from the research phase to prove the source of the data?*

**Response**: Thank you for your suggestion. We have taken pictures from the research phase to prove the source of the data, and we included them with the Supporting Information. However, some pictures may not have been added due to the copyright issues. Your feedback is much appreciated.

*Comment18:Is there an intuitive picture that describes the content of this article?*
**Response**: Regarding your question about an intuitive picture that describes the content of our article, we appreciate your interest in visual representation. While our article primarily focuses on textual content, we do not have a specific intuitive picture related to the subject matter. However, we have incorporated relevant figures, table within the article and Supporting Information to enhance the understanding of the presented data and findings. These visual aids serve to complement and support the written information.

*Comment19:The necessary cultural background is not stated, and as an international journal, this can affect readers' understanding. There are too many topics that people in other countries and regions do not understand, so they should be more specific*
**Response**: Thank you for your comment regarding the cultural background in our manuscript. We acknowledge the importance of providing sufficient cultural context for international readers. We would like to clarify that due to the limited availability of literature on the cultural background of the Bede snake charmers in Bangladesh, it is indeed challenging to provide extensive details in this regard. However, we have made efforts to include relevant information wherever possible to facilitate readers' understanding. We appreciate your understanding of the constraints and will continue to strive for clarity in presenting our research findings.

*Comment20:1re the people included in the study a single factor? Or is it influenced by more factors? This is related to the reliability of this paper. The authors did not report specific analyses, and the conclusions are far-fetched.*
**Response**: We appreciate your feedback and concerns regarding the factors influencing the health seeking behavior of the Bede people in our study. We agree that it is essential to consider multiple factors rather than relying on a single factor alone.

In our research, we conducted in-depth interviews and qualitative analyses to explore the barriers faced by the Bede people when seeking hospital treatment for snakebites. The aim was to provide a comprehensive understanding of their health seeking behavior. Through this process, we identified four key barriers that emerged from the interviews: (1) geographic accessibility, (2) affordability, (3) availability, and (4) acceptability of hospital treatment.

The inclusion of these four barriers was based on a rigorous analysis of the interview data, and we have made sure to report these findings accurately in our manuscript. We understand the importance of providing specific analyses and discussing them in detail. Therefore, in the revised version of the paper, we will provide more specific information on the analysis techniques employed and the process through which these barriers were identified.

By considering these multiple factors, we aim to present a comprehensive picture of the challenges faced by the Bede people and the complexities surrounding their health seeking behavior. We appreciate your comment, and we will make the necessary revisions to enhance the clarity and reliability of our paper. Thank you for your valuable input.

*Comment21: How were the conclusions reached when the authors did not analyze the data at all?*

**Response**: Thank you for your comment and concern. We would like to clarify that we did analyze the data in our study using conventional content analysis. This method allowed us to systematically examine the qualitative data and identify key themes and patterns related to the health seeking behavior of the Bede people regarding snakebite treatment. Through this analysis, we were able to identify and categorize the four barriers: (geographic) accessibility, affordability, availability, and acceptability. The conclusions presented in our manuscript were reached based on the findings from this analysis process. We acknowledge the importance of rigorous data analysis in research, and we assure you that our study followed appropriate analytical procedures to derive meaningful conclusions.

*Comment22: How do I prove it? The problem is too serious.*
**Response**: We understand your concern regarding the seriousness of the problem addressed in our study. We would like to clarify that although qualitative research may not provide quantifiable measures of proof in the same way as quantitative research, it offers valuable insights and understanding of complex phenomena. In our study, we employed a qualitative approach to explore and understand the health seeking behavior of the Bede people regarding snakebite treatment. Through in-depth interviews and the use of conventional content analysis, we analyzed the data collected from various sources, including Bede individuals who had experienced snakebites, their family members, Bede snakebite healers, and key informants. By utilizing rigorous qualitative research methods, including interviews conducted by trained researchers and the systematic analysis of data, we aimed to provide a comprehensive understanding of the barriers faced by the Bede community in seeking hospital treatment for snakebite. Our findings revealed four key barriers: (geographic) accessibility, affordability, availability, and acceptability. While our study may not prove causation or establish statistical significance in the traditional sense, it contributes to the existing body of knowledge by shedding light on the factors that influence health seeking behavior in a specific cultural context. The conclusions drawn from our qualitative analysis are based on the patterns and themes identified through rigorous data analysis and triangulation. We acknowledge the limitations of our study, including the difficulty in reproducibility inherent in qualitative research. However, we have taken steps to enhance transparency and reproducibility by following the COREQ guidelines, which provide a structured framework for reporting qualitative research methods. In conclusion, we appreciate your concern, and we want to assure you that our study utilized robust qualitative research methods to analyze the data and draw conclusions. While qualitative research may not offer the same type of proof as quantitative research, it provides valuable insights into complex social phenomena. We believe that our study contributes to the understanding of health seeking behavior among the Bede people and serves as a foundation for further research in this area.

*Comment23: Concepts are not clearly defined in the last few sections of the manuscript.*
**Response**: Thank you for your comment regarding the clarity of concepts in the last few sections of our manuscript. We appreciate your feedback and understand the importance of clearly defining and explaining concepts to ensure the reader's understanding.

Upon reviewing the sections in question, we believe that the concepts related to the health seeking behavior of the Bede people and their treatment options for snakebite are adequately defined and explained. We discuss the barriers faced by the Bede people, including accessibility, affordability, availability, and acceptability of treatment, and how these factors influence their health seeking behavior. Additionally, we provide insights into the Bede's beliefs in herbal medicine, such as the Indian snakeroot, and its availability.

We acknowledge that there may be room for further improvement in terms of clarity, and we carefully reviewed and revised the manuscript to ensure that the concepts are presented in a more explicit and understandable manner.

*Comment24:Conclusions are arbitrary, lack evidence, and need to be supplemented.*
**Response**:

Thank you for your valuable comment. We revised as follows.

| Original version | Revised version |
| --- | --- |
| In conclusion, only one successful anti-venom treatment was reported in this study. While many Bede people operate in the snake business, and snakebite is a common occupational hazard among them, accessibility to anti-venom treatment is a barrier for the Bede community. The Bede’s traditional treatments are less effective while many of them rely on their traditional treatment, specially their herbal medicine. Consequently, a few Bede people die of snakebite every year and many others suffer from suffer severe consequences. To make the anti-venom treatment available within/near the community, collaborations between modern medicine in the public sector and the traditional medicine of Bede should be promoted. Training Bede traditional healers on modern treatment and use of anti-venom and deploying the trained Bede people at rural medical facilities are also recommended. The WHO’s snakebite program as an NTD control should be practical so that marginalized people such as the Bede are also included. | In conclusion, the Bede community faces significant challenges in accessing timely and appropriate snakebite treatment. Limited successful antivenom cases have been reported, and the community faces barriers in accessibility, affordability, availability, and acceptability of treatment. Traditional treatments are less effective and can lead to fatalities and severe consequences. Collaborations between modern and traditional medicine, training of Bede healers, and inclusion in snakebite programs are recommended to address these challenges. |

*Comment25: The contrast is confusing, and I don't know what I want to say.*
**Response**: Thank you for your feedback regarding the contrast in our manuscript. We apologize if the comparison or contrast presented in the paper was confusing or unclear. We understand the importance of providing clear and coherent explanations to facilitate understanding.

To address this concern, we carefully reviewed the manuscript and identified areas where the contrast may not be adequately explained or may be causing confusion so that revise the text to provide clearer explanations and ensure that the intended message is effectively conveyed. Additionally, we considered using visual aids, such as tables or figures, to enhance the clarity of the contrasts and comparisons presented in the paper.

*Comment26:The author completely failed to face up to and modify the comments and suggestions of the previous reviewers and only made some modifications unrelated to the center, which is meaningless. There seems to be a problem with the author's academic attitude.*

**Response**:

We appreciate your feedback and concerns regarding our response to the previous reviewers' comments. We apologize if it gave the impression that we did not adequately address the suggestions and modifications. We would like to clarify that we carefully considered and incorporated the comments from the reviewers in the revised version of the manuscript.

We understand the importance of addressing the core issues raised by the reviewers and ensuring that the modifications are meaningful and relevant to the study. While it may not have been explicitly mentioned in our response, we assure you that we have taken all the comments seriously and made necessary revisions to improve the manuscript.

We value the feedback provided by the reviewers and other experts in the field, and we have made every effort to enhance the quality and clarity of our research. We take our academic responsibilities seriously and strive to maintain a high standard in our work.

We are also grateful for the positive feedback from Reviewer #2 and Reviewer #3, who found the revisions satisfactory and acknowledged the improvements made to the manuscript. We believe that the revisions have strengthened the study and addressed the reviewers' comments.

We appreciate your input and will carefully consider your comments to further improve the manuscript if necessary. Thank you for your valuable feedback.

*Comment27: The contents of nationality disputes should be properly handled.*
Response: Thank you for your valuable feedback on our manuscript. We genuinely appreciate your comment regarding the handling of nationality disputes in our study. We would like to clarify that our research has a specific focus on the health seeking behavior of the Bede people regarding snakebite, and it does not directly address nationality disputes. Our objective is to clarify the health seeking behavior of the Bede people regarding snakebite and its outcome. Nationality disputes fall outside the scope of our study, and we have made sure to maintain our focus on the intended objective, providing valuable insights into the health seeking behavior of the Bede people.

Furthermore, we acknowledge the limitation of our study, which is the limited literature available on the Bede people, who are the participants of our research. Due to this limited literature, addressing or delving into issues related to nationality disputes becomes challenging. Nevertheless, we have made diligent efforts to provide insights based on the available data and participant accounts. We recognize that further research and exploration are necessary to gain a comprehensive understanding of the complexities surrounding nationality disputes among the Bede people.

*Comment28: In conclusion, this manuscript is too crude and needs to be revised. It should not even be accepted for publication.*

**Response**: Thank you for your feedback on our manuscript. We appreciate your comments regarding the need for revision. We acknowledge that there is room for improvement in the current version of the manuscript. We want to assure you that we have taken your feedback seriously and have made significant efforts to address the issues raised by the reviewers. The manuscript has undergone extensive revisions to enhance its clarity, coherence, and overall quality. We believe that these revisions have substantially improved the manuscript and have addressed many of the concerns raised. However, we acknowledge that there may still be areas that require further refinement. We are committed to continuously revising and enhancing the manuscript to meet the highest academic standards. We value your input and would appreciate any specific suggestions or areas of concern that you believe require further attention. Your guidance will greatly assist us in further improving the revised manuscript. Once again, we appreciate your feedback and the opportunity to enhance our work.
